# A High-Precision Inverse Finite Element Method for Shape Sensing and Structural Health Monitoring

**DOI:** 10.3390/s24196338

**Published:** 2024-09-30

**Authors:** Hongsheng Yan, Jiangpin Tang

**Affiliations:** School of Civil Engineering, Tianjin University, Tianjin 300350, China; holmes_tj@126.com

**Keywords:** iFEM, shape sensing, structural health monitoring

## Abstract

In the contemporary era, the further exploitation of deep-sea resources has led to a significant expansion of the role of ships in numerous domains, such as in oil and gas extraction. However, the harsh marine environments to which ships are frequently subjected can result in structural failures. In order to ensure the safety of the crew and the ship, and to reduce the costs associated with such failures, it is imperative to utilise a structural health monitoring (SHM) system to monitor the ship in real time. Displacement reconstruction is one of the main objectives of SHM, and the inverse finite element method (iFEM) is a powerful SHM method for the full-field displacement reconstruction of plate and shell structures. However, existing inverse shell elements applied to curved shell structures with irregular geometry or large curvature may result in element distortion. This paper proposes a high-precision iFEM for curved shell structures that does not alter the displacement mode of the element or increase the mesh and node quantities. In reality, it just modifies the methods of calculation. This method is based on the establishment of a local coordinate system on the Gaussian integration point and the subsequent alteration of the stiffness integration. The results of numerical examples demonstrate that the high-precision iFEM is capable of effectively reducing the displacement difference resulting from inverse finite element method reconstruction. Furthermore, it performs well in practical engineering applications.

## 1. Introduction

Today, as deep-sea resources continue to be exploited, ships play an important role in many aspects, such as offshore oil and gas extraction, pelagic fishing, and scientific research [1,2,3]. However, ships are often exposed to harsh marine environments. Ship structures can fail due to extreme or cyclic loading, corrosion, and erosion. Structural failure can lead to major accidents resulting in the loss of life of crew or passengers, pollution of the marine environment, and high maintenance or repair costs [4,5]. Therefore, it is imperative to utilise a structural health monitoring (SHM) system to monitor the ship in real time. Structural Health Monitoring (SHM) [6] is an interdisciplinary process that integrates a sensing system on a structure and processes the data collected by the sensing system in real time to provide critical real-time information on the overall or local structural health of the structure. 

For many years, different types of ships have applied different ship monitoring methods. In 1998, Kageyama et al. [7] developed a fibre laser Doppler velocimeter displacement sensor for measuring the global deformation of marine structures. In 2005, Torkildsen et al. [8] outlined a ship health monitoring system installed on Royal Norwegian Navy vessels, which can provide real-time feedback on overall wave loads on the ship and local loads in many selected critical areas. In 2011, Andersson et al. [9] proposed a ship condition monitoring system for the damage monitoring of ships made of fibre-reinforced plastics. In 2012, Sielski [10] developed full-life-cycle performance analysis and monitoring technology for aluminium ship structural systems using a ship reliability programme that includes structural health monitoring tools.

In fact, current ship monitoring systems only monitor a few points on the structure, and these studies have not evaluated the strain and stress conditions between monitoring points that may experience maximum strain. Due to the complex topology of marine structures and the complexity and statistical characteristics of marine phenomena, the global structural monitoring of ships is more challenging than existing ship monitoring systems. By reconstructing the three-dimensional full field displacement of the structure, this challenge could be addressed [11,12,13].

At present, most displacement reconstruction methods such as the Amongst Model Method [14] and Ko’s Displacement Theory [15] require either property information about structural materials or sufficiently accurate load information. However, due to the complexity and statistical characteristics of the marine environment, estimating the dynamic loads of wind and waves is not easy. In addition, some methods are limited to static loads and linear displacements due to their inherent assumptions, and most methods are time-consuming for analysis, which is not fast enough for feasible real-time applications [16]. Therefore, these methods are not suitable for use in ship real-time monitoring systems. The algorithm that best fits the above required features is the inverse finite element method (iFEM). The iFEM is one of the structural health monitoring methods. In practical engineering applications, it is used to reconstruct the overall displacement field of the structure in real time by collecting strain data from discrete points on the structure, realising the conversion from strain to displacement. Thus, the overall deformation information of the structure is obtained, and then the real-time safety assessment of the structural state is carried out [17].

Tessler and Spangler [18] developed the inverse finite element method (iFEM) for the shape sensing of engineering structures. In contrast to other developed SHM methods, iFEM is universally applicable to any type of structure and boundary condition because the physical domain can be efficiently discretised using inverse beam, plate, and shell elements. Since the iFEM weighted least squares function is only determined through the strain–displacement relationship, the iFEM allows the reconstruction of the deformed shape of a structure without prior knowledge of the material properties and loading information. Additionally, unlike other SHM systems, the stability and accuracy of the shape-sensing results obtained by the iFEM are independent of any type of static or dynamic loading acting on the structure. Once the deformed shape of the structure is obtained, the full-field displacement can be calculated from the strain–displacement relationship, and, thus, the full-field stress of the structure can be evaluated. 

Tessler and Hughes [19] developed a three-node inverse shell element (iMIN3) using first-order kinematic assumptions and shear deformation theory. Quach and Vazquez et al. [20] confirmed the stability of the iMIN3 element by experimentally measuring real-time strain data. Kefal et al. [21,22] developed a four-node quadrilateral inverse shell element iQS4 and an eight-node quadrilateral inverse shell element iCS8 based on Mindlin plate theory and degradation shell theory. In addition, Kefal and Oterkus [23,24] performed the deformation monitoring of plates and shells as a basic application of the iFEM in the SHM of marine structures. Kefal and Oterkus [25,26] presented a more sophisticated application of iFEM in marine structures. Li and Kefal et al. [27] performed the deformation monitoring of a submarine pressure hull.

In recent years, there have been many new developments in the inverse finite element method. Li and Oterkus et al. [28] presented a two-dimensional eight-node quadrilateral inverse finite element formulation that is suitable for thin structures under in-plane loading conditions. Kefal et al. [29] successfully utilised iFEM for the monitoring of offshore wind turbine blades. Zhu and Wu et al. [30] presented a groundbreaking approach to strain field reconstruction in such panels. Colombo et al. [31] explored an inverse finite element method based on effect superposition. Esposito and Roy et al. [32] presented a novel methodology for the accurate and efficient elastic deformation reconstruction of thin-walled and stiffened structures from discrete strains. Roy and Tessler et al. [33] presented a novel SEA-iFEM approach that can obtain accurate shape-sensing reconstructions for plate structures even with a small number of strain sensors and a judicial choice of strain sensor layouts. Kefal et al. [34] developed a novel structural health monitoring approach by coupling iFEM and peridynamic theory (PD) for real-time shape sensing analysis and the crack propagation monitoring of plate structures. Zhu and Du et al. [35] put forward a displacement reconstruction approach based on limited strain data using iFEM. Abdollahzadeh and Ali et al. [36] demonstrated the high efficiency, robustness, and accuracy of the iFEM approach in reconstructing geometrically non-linear deformations of thin laminated plates and shells. Zhao and Kefal et al. [37] proposed a novel shape-sensing method for large deformation monitoring based on strain gradient theory and iFEM.

In 2016, a four-node quadrilateral inverse shell element (iQS4) was first applied to thin-walled cylinder shells [21], which demonstrated the potential for a quadrilateral inverse shell element to be used in three-dimensional structures and provided initial experience. Nevertheless, the utilisation of the conventional iQS4 element to analyse curved shell structures does not ensure that all of the nodes of the element are situated in the same plane, which gives rise to the warping issue of the element and precludes the accurate simulation of the surface geometry [38]. Consequently, the mesh must be refined in order to approximate the genuine surface shape. In particular, for curved shell structures with irregular geometry or considerable curvature, a dense mesh is required to achieve satisfactory accuracy, which consequently entails a considerable amount of computation [38]. Furthermore, although the curved shell element is capable of accurately representing the shape of a curved shell, the challenge lies in accurately expressing the rigid body motion to avoid shear and membrane locking, which makes the construction of the curved shell element a complex process [39]. 

A variety of large-scale marine structures, including ship hulls, onshore and offshore structures, and submarines, are all marine structures with complex curved surfaces [22]. To enhance the computational precision of the iQS4 element in these structures, a novel iFEM algorithm is presented. Firstly, this article proposes a methodology for establishing a local coordinate system at Gaussian integration points based on the iQS4 element and introduces a novel calculation method for element stiffness integration. Subsequently, a novel algorithm is devised and programmed as a computer program. To comprehensively assess the efficacy of the proposed algorithm in the deformation reconstruction of curved shell structures, two representative examples are selected for numerical analysis and compared with the conventional iQS4 element to ascertain its accuracy and convergence rate. Furthermore, to ascertain the engineering viability of the proposed algorithm, a numerical analysis is conducted on a representative containership. This analysis aims to ascertain the algorithm’s ability to accurately reconstruct the deformation of marine structures with complex surfaces.

## 2. Methods of Calculation

**Figure 1:**
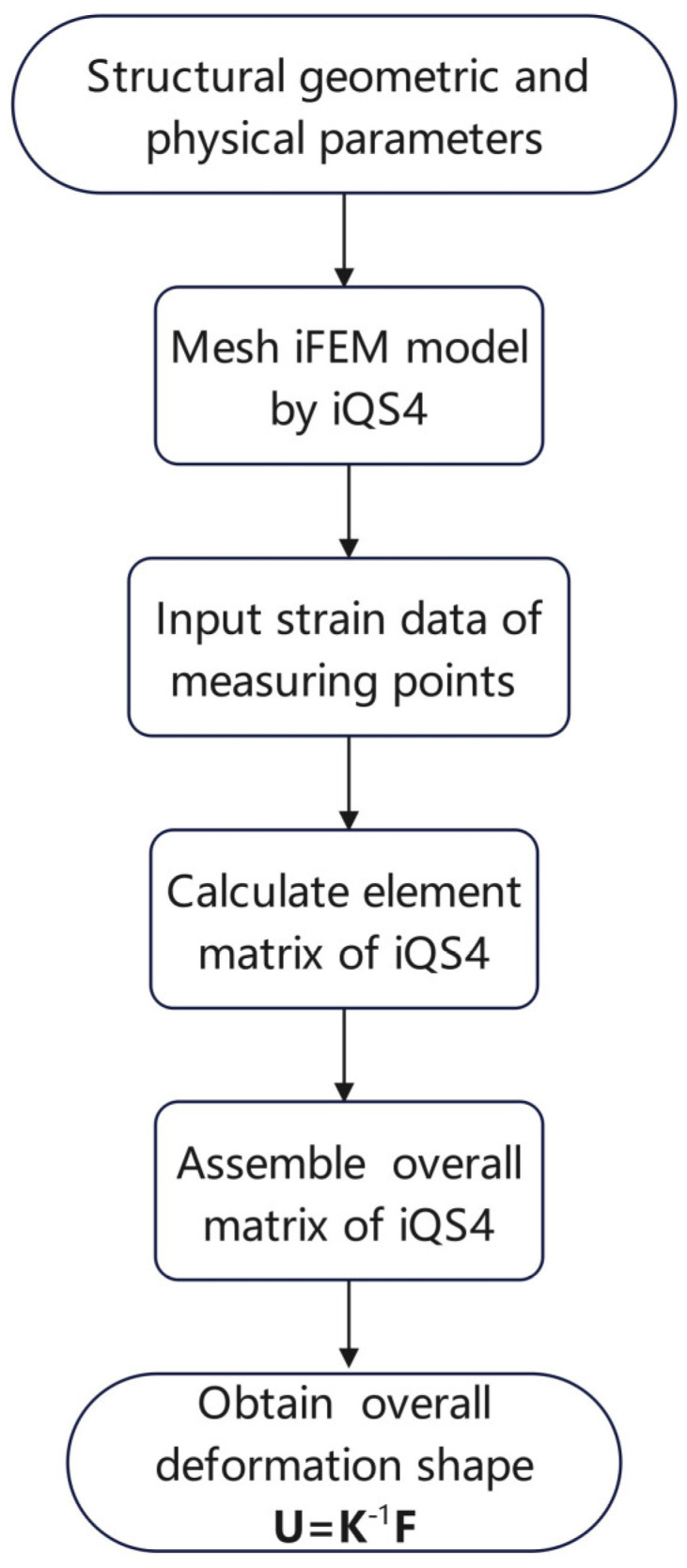
Implementation process of the iQS4 element.

In order to specifically analyse the limitations of the conventional iFEM algorithm, we present the implementation process of the iQS4 element (shown in Figure 1).

Compared with the conventional iFEM algorithm, we propose several new calculation methods for steps 4 and 5 (shown in Figure 1). Firstly, we propose a novel method for establishing a local coordinate system.

### 2.1. Methods for Establishing Local Coordinate System

**Figure 2:**
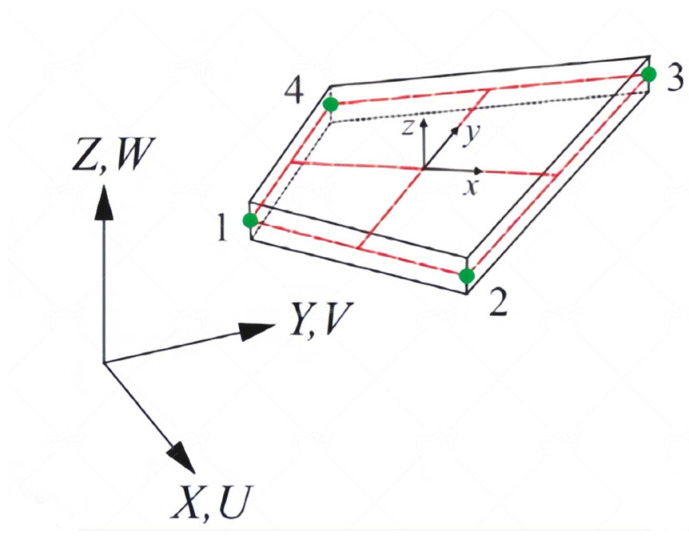
Local coordinate system of the iQS4 element.

Firstly, the conventional method for establishing a local coordinate system is introduced (shown in Figure 2). The local coordinates of the inverse element are defined as (x,y,z), and the global coordinates of the inverse element are defined as (X,Y,Z). Subsequently, the local coordinates of the element can be converted to the global coordinates by means of a suitable transformation matrix, which, in turn, assembles the element matrix into a global matrix.

**Figure 3:**
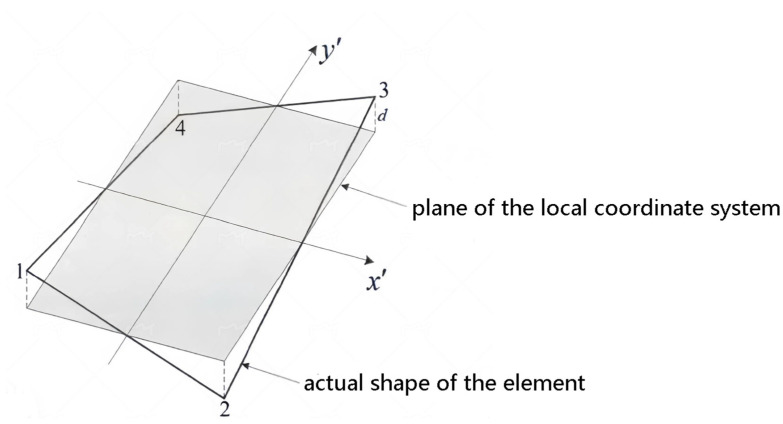
Geometric representation of a warped iQS4 element.

In the process of applying the iQS4 element to the meshing of curved structures, particularly those with irregular geometry or large curvature, it is possible that the four nodes of the element may not be in the same plane. For such a warped element, the local coordinate system of the element established by the conventional method is inadequate for accurately describing the position information of the element. This is particularly evident when the element stiffness matrix is converted from the local coordinate system to the overall coordinate system, which results in a significant discrepancy. As illustrated in Figure 3, due to the fact that the four nodes of the element are not situated in the same plane, the plane where the local coordinate system of the element is situated and the surface of the element are not in complete alignment. The degree of discrepancy can be quantified by the distance D between the nodes.

In order to overcome the limitations of the iQS4 element, a method for establishing a local coordinate system on the tangent plane of the element is presented. For ease of presentation, the coordinate identifiers of overall coordinates (X,Y,Z) are written as $\left(x_{1},x_{2},x_{3}\right)$, the coordinate identifiers of the local coordinates (x,y,z) are written as $\left({x_{1}}^{\prime },{x_{2}}^{\prime },{x_{3}}^{\prime }\right)$, and the coordinate identifiers of the parameter coordinates are still (ξ,η).

The key to establishing the local coordinate system on the element tangent plane is to determine the tangent plane of the element at the origin. Initially, the identification of the bilinear isoparametric shape function N(ξ,η) of the four-node quadrilateral element is requisite. Subsequently, it is necessary to identify the coordinates within the parametric coordinate system$—x_{1}(\xi ,\eta )$, $x_{2}(\xi ,\eta )$, $x_{3}(\xi ,\eta )$—which can be expressed in terms of the coordinates of each node of the element in the overall coordinate system:

(1) $$x_{i}(\xi ,\eta )=\sum_{a=1}^{M}N_{a}(\xi ,\eta )x_{i}^{a}\;(i=1\sim 3)$$

**Figure 4:**
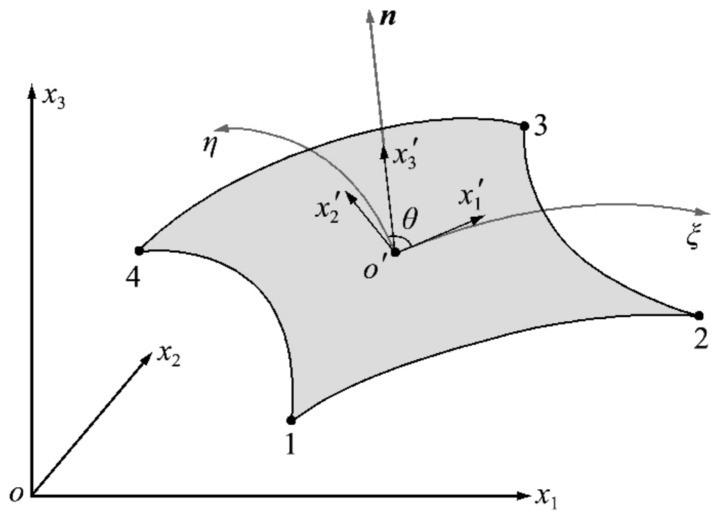
Tangent plane local coordinate system.

$N_{a}(\xi ,\eta )$ is the form function of the element. M is the number of element nodes for iQS4 element M=4. Once the parametric coordinate system has been established, the next step is to define the local coordinate system at the element tangent plane. The origin of the parametric coordinate system is taken as the origin of the local coordinate system, the direction of tangent to the ξ axis of the parametric coordinate system is taken as the ${x_{1}}^{\prime }$ direction, and the direction of normal to the element surface as the ${x_{3}}^{\prime }$ direction. According to the right-hand rule, the ${x_{2}}^{\prime }$ direction is then determined. The ${x_{1}}^{\prime }o^{\prime }{x_{2}}^{\prime }$ plane where the local coordinate system of the element is located is the tangent plane of the shell element at the origin position $o^{\prime }$, as shown in Figure 4.

Once the tangent plane local coordinate system has been established, the next step is to obtain the unit vectors $r_{\xi }{,r}_{\eta }$, and n characterising the directions of the three coordinate axes, which are perpendicular to each other. According to the literature [40], the expression is:

(2a) $${r_{\xi }}^{\prime }=\frac{\partial x_{1}(\xi ,\eta )}{\partial \xi }i+\frac{\partial x_{2}(\xi ,\eta )}{\partial \xi }j+\frac{\partial x_{3}(\xi ,\eta )}{\partial \xi }k$$

(2b) $${r_{\eta }}^{\prime }=\frac{\partial x_{1}(\xi ,\eta )}{\partial \eta }i+\frac{\partial x_{2}(\xi ,\eta )}{\partial \eta }j+\frac{\partial x_{3}(\xi ,\eta )}{\partial \eta }k$$

By unitising ${r_{\xi }}^{\prime }$, ${r_{\eta }}^{\prime }$, and $n^{\prime }$, the unit vectors $r_{\xi }$, $r_{\eta }$, and n characterising the directions of the three coordinate axes have been derived. As a result, according to the literature [41], the coordinate transformation matrix $T^{e}$ for the tangent plane local coordinate system can be expressed as:

(3a) $${T^{e}}_{1j}=\frac{1}{\left|m_{1}\right|}\frac{\partial x_{j}}{\partial \xi }\;(j=1\sim 3)$$

(3b) $${T^{e}}_{21}={{n_{2}T^{e}}_{13}-{n_{3}T^{e}}_{12}}_{,}$$

(3c) $${T^{e}}_{22}={{n_{3}T^{e}}_{11}-{n_{2}T^{e}}_{13}}_{,}$$

(3d) $${T^{e}}_{23}={{n_{1}T^{e}}_{12}-{n_{2}T^{e}}_{11}}_{,}$$

(3e) $${T^{e}}_{3j}=n_{j}(j=1\sim 3)\;\;$$

$\left|m_{1}\right|$ is the module of $r_{\xi }$, $\left|m_{2}\right|$ is the module of $r_{\eta }$.

At this point, the stiffness transformation matrix T of the element can be determined. For a four-node shell element, T is a 24×24 matrix, which can be expressed as:

(4) $$T=\left[\begin{array}{c} \begin{array}{ccc} T^{e} & 0 & \begin{array}{ccc} 0 & 0 & \begin{array}{ccc} 0 & 0 & \begin{array}{cc} 0 & 0 \end{array} \end{array} \end{array} \end{array}\\ \begin{array}{c} \begin{array}{ccc} 0 & T^{e} & \begin{array}{ccc} 0 & 0 & \begin{array}{ccc} 0 & 0 & \begin{array}{cc} 0 & 0 \end{array} \end{array} \end{array} \end{array}\\ \begin{array}{c} \begin{array}{ccc} 0 & 0 & \begin{array}{ccc} T^{e} & 0 & \begin{array}{ccc} 0 & 0 & \begin{array}{cc} 0 & 0 \end{array} \end{array} \end{array} \end{array}\\ \begin{array}{ccc} 0 & 0 & \begin{array}{ccc} 0 & T^{e} & \begin{array}{ccc} 0 & 0 & \begin{array}{cc} 0 & 0 \end{array} \end{array} \end{array} \end{array}\\ \begin{array}{c} \begin{array}{ccc} 0 & 0 & \begin{array}{ccc} 0 & 0 & \begin{array}{ccc} T^{e} & 0 & \begin{array}{cc} 0 & 0 \end{array} \end{array} \end{array} \end{array}\\ \begin{array}{ccc} 0 & 0 & \begin{array}{ccc} 0 & 0 & \begin{array}{ccc} 0 & T^{e} & \begin{array}{cc} 0 & 0 \end{array} \end{array} \end{array} \end{array}\\ \begin{array}{c} \begin{array}{ccc} 0 & 0 & \begin{array}{ccc} 0 & 0 & \begin{array}{ccc} 0 & 0 & \begin{array}{cc} T^{e} & 0 \end{array} \end{array} \end{array} \end{array}\\ \begin{array}{ccc} 0 & 0 & \begin{array}{ccc} 0 & 0 & \begin{array}{ccc} 0 & 0 & \begin{array}{cc} 0 & T^{e} \end{array} \end{array} \end{array} \end{array} \end{array} \end{array} \end{array} \end{array} \end{array}\right]$$

The element stiffness matrix $k^{e}$ is converted to the overall coordinate system by means of the stiffness transformation matrix T to obtain K:

(5) $$K=T^{T}(\sum_{i=1}^{N}\sum_{j=1}^{N}\varphi_{ij})T$$

N denotes the number of Gaussian integration points; $\varphi_{ij}$ is the calculated value of the element stiffness matrix at the Gaussian integration points. 

As previously stated, there is a discrepancy between the local coordinate system of the iQS4 element and the element surface. This can be quantified by the distance D between the nodes. To enhance the computational accuracy, it is essential to reduce this distance D.

**Figure 5:**
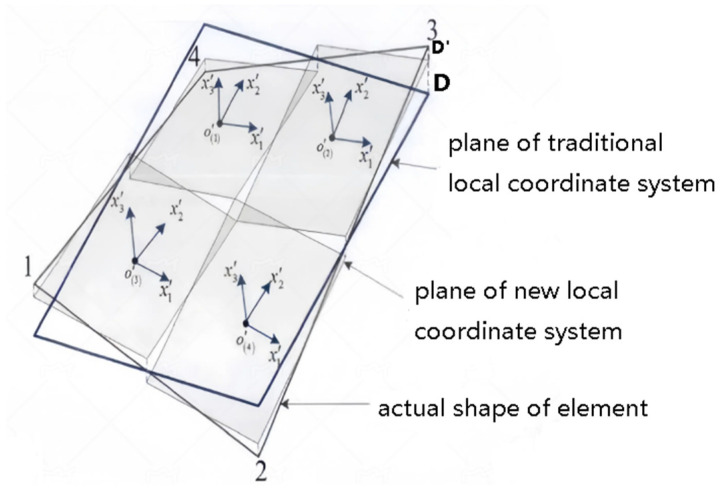
Comparison of two methods for establishing a local coordinate system.

As stated in the literature [42], the Gaussian integration summation process only considers values at the Gaussian integration points. Consequently, a distinct local coordinate system is established at each integration point, which ensures the accuracy of each summation process and stiffness conversion process. The establishment of a local coordinate system on the tangent plane at the Gaussian integration point of the element surface ensures that the application of multiple local coordinate systems can effectively reflect the geometry of the surface shell and reduce the distance D, as illustrated in Figure 5.

**Figure 6:**
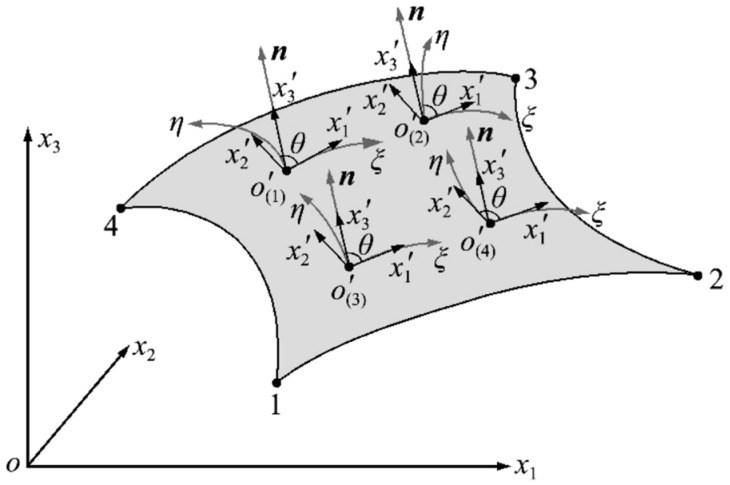
Local coordinate system established at Gaussian integration points.

To illustrate, the 2×2 exact Gaussian integration is considered as an example. In this case, multiple local coordinate systems are established, with each Gaussian point serving as the origin. Each local coordinate system is established in a manner analogous to the previous local coordinate system, with the exception that the coordinate origin is set at the Gaussian integration points, as showed in Figure 6.

In the local coordinate system at each Gaussian integration point, the coordinate transformation matrix from the local coordinate system to the overall coordinate system is obtained according to Equations (3) and (4), and the stiffness value at each Gaussian integration point is converted to the overall coordinate system before the integration and summation. Depending on the position of the tangent plane where the local coordinate system is located, the conversion matrix is corrected in time. The expression of the stiffness matrix in the overall coordinate system should be modified as:

(6) $$K=\sum_{i=1}^{N}\sum_{j=1}^{N}T_{ij}^{T}\varphi_{ij}T_{ij}$$

N denotes the number of Gaussian integration points; $T_{ij}$ denotes the stiffness transformation matrix from the local coordinate system established at different Gaussian integration points to the overall coordinate system; and $\varphi_{ij}$ denotes the calculated value of the element stiffness matrix at the Gaussian integration points.

In theory, the greater the number of Gaussian integration points associated with an element, the more effective the reduction in the distance D will be. For sparser meshing, given that D is considerable, it is possible to utilise a greater number of Gaussian integration points in order to achieve a significant reduction in the distance D. However, for denser meshing, the magnitude of D may be relatively small, and the addition of corrections to the transformation matrix at the Gaussian integration points will result in a certain amount of additional computational load. Consequently, the use of an excessive number of Gaussian integration points may result in a significant increase in the computational load.

**Figure 7:**
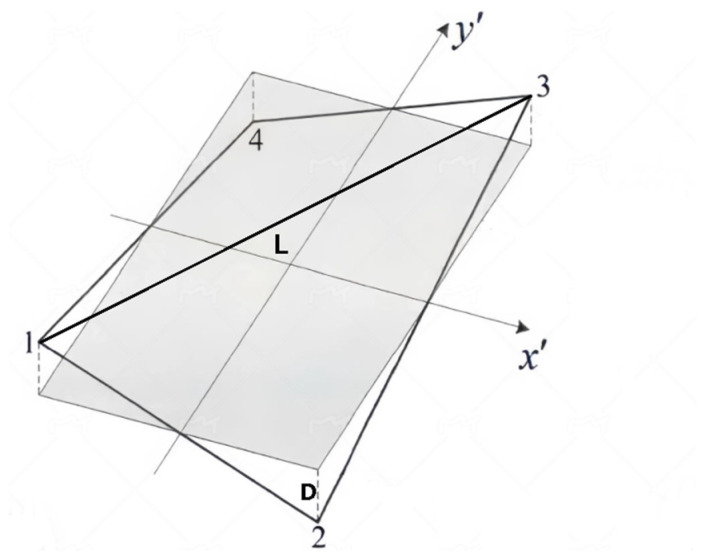
Geometric meaning of D and L.

To balance the computational load with the distance D between nodes to ensure optimal accuracy within an acceptable range, it is necessary set a convergence criterion. In this paper, the diagonal length corresponding to each node is defined as L, as shown in Figure 7. The ratio of D to L, which can be expressed as D/L, is defined as the warpage rate W.

According to the literature [38], if the maximum W of the element is a more than acceptable value, which is about 5%, it can be assumed that the element warpage should be corrected. Subsequently, the 2×2 Gaussian integration is initially employed to calculate the W of each node for each element. If the maximum W of the element is less than 1%, then it can be considered to have reached the convergence criterion. If the maximum is more than 1%, continue to use the 3×3 Gaussian integration to calculate W, and so on, until reaching the convergence criterion.

### 2.2. Methods for Calculating Element Stiffness Integration

According to the iFEM formulation, the element matrix equation for the iQS4 element is as follows:

(7) $$k^{e}u^{e}=f^{e}$$

where $k^{e}$ is the element stiffness matrix and $f^{e}$ is the element load column matrix, which can be calculated from Equation (8):

(8a) $$k^{e}=\iint (w^{m}(B^{m}{)}^{T}B^{m}+w^{b}(2h{)}^{2}(B^{b}{)}^{T}B^{b}+w^{g}(B^{g}{)}^{T}B^{g})dxdy$$

(8b) $$f^{e}=\iint (w^{m}(B^{m}{)}^{T}e_{i}^{\varepsilon }+w^{b}(2h{)}^{2}(B^{b}{)}^{T}k_{i}^{\varepsilon }+w^{g}(B^{g}{)}^{T}g_{i}^{\varepsilon })dxdy$$

where $e_{i}^{\varepsilon }{{,k}_{i}^{\varepsilon },g}_{i}^{\varepsilon }$ are strains measured by the strain transducer, $w^{m},\;\;w^{b}$, and $w^{g}$ are the weighting constants, and $B^{m}$, $B^{b}$, and $B^{g}\;$ are the element strain matrices. 

To obtain the element matrices $k^{e}$ and $f^{e}$, it is necessary to calculate the derivatives of the element shape functions with respect to the local coordinates. The conventional iFEM requires the derivation of the transformation relationship between the partial derivatives $\frac{\partial }{\partial x},\frac{\partial }{\partial y}$ and $\frac{\partial }{\partial \xi },\frac{\partial }{\partial \eta }$ as well as the computation of the Jacobi determinant $\left|J\right|$, which can be calculated according to the law of derivation of composite functions:

(9a) $$\left[\begin{array}{c} \frac{\partial }{\partial x}\\ \frac{\partial }{\partial y} \end{array}\right]={\left[J\right]}^{-1}\left[\begin{array}{c} \frac{\partial }{\partial \xi }\\ \frac{\partial }{\partial \eta } \end{array}\right]$$

(9b) $$\left[J\right]=\left[\begin{array}{cc} \frac{\partial x}{\partial \xi } & \frac{\partial y}{\partial \xi }\\ \frac{\partial x}{\partial \eta } & \frac{\partial y}{\partial \eta } \end{array}\right]=\left[\begin{array}{cc} \sum_{i=1}^{4}\frac{\partial N_{i}}{\partial \xi }x_{i} & \sum_{i=1}^{4}\frac{\partial N_{i}}{\partial \xi }y_{i}\\ \sum_{i=1}^{4}\frac{\partial N_{i}}{\partial \eta }x_{i} & \sum_{i=1}^{4}\frac{\partial N_{i}}{\partial \eta }y_{i} \end{array}\right]$$

$x_{i}$ and $y_{i}$ are the coordinate values obtained after converting the overall coordinates of the element to the two-dimensional plane local coordinate system. 

The difference in iQS4 largely originates from the distance D between the plane where the local coordinate system is located and the element surface [42]. In order to reduce D, this article introduces a method for calculating shape function derivatives in a three-dimensional global coordinate system. $N_{i,x^{\prime }},N_{i,y^{\prime }},$ and Jacobi determinant $\left|J\right|$ are derived without converting the overall coordinates to the two-dimensional local coordinates. From the formula derived by Gao and Davies [40], the Jacobi determinant $\left|J\right|$ for the conversion from overall coordinates to parameter coordinates is given as:

(10) $$\left|J\right|=\left|r_{\xi }\times r_{\eta }\right|$$

$r_{\xi }$, $r_{\eta }$ are vectors tangent to the parametric coordinate axes $\left(\xi ,\eta \right),\;$respectively, at the origin. From the law of derivation of a composite function, we know that:

(11) Ni,x′Ni,y′=Ni,x1′Ni,x2′=∂ξ∂x1′∂η∂x1′∂ξ∂x2′∂η∂x2′Ni,ξNi,η

The formulae derived by Lachat and Becher are given as [43,44]:

(12) $${{\;\;x}_{1}}^{\prime }=\left|m_{1}\right|\xi +\left|m_{2}\right|\eta cos\theta$$

(13) $${{\;x}_{2}}^{\prime }=\left|m_{2}\right|\eta sin\theta$$

θ is the angle between the ${x_{1}}^{\prime }$ axis and η axis shown in Figure 3.

Once the element stiffness matrix $B^{m}$, $B^{b}$ and $B^{g}$ are calculated, the overall stiffness matrix K and the overall load column matrix  F can be synthesised according to the method of assembling the overall stiffness matrix described in the previous section. Finally, the overall displacement U can be obtained.

### 2.3. Algorithm Implementation

In this section, the method of establishing the local coordinate system at Gaussian integration points is proposed and a method for the inverse shell element stiffness integration is introduced. This is, in fact, a new computational approach to iFEM, which does not affect the displacement mode of the element. For the convenience of the study, this thesis is dedicated to the problem of small deformation of the static force only in this paper.

**Figure 8:**
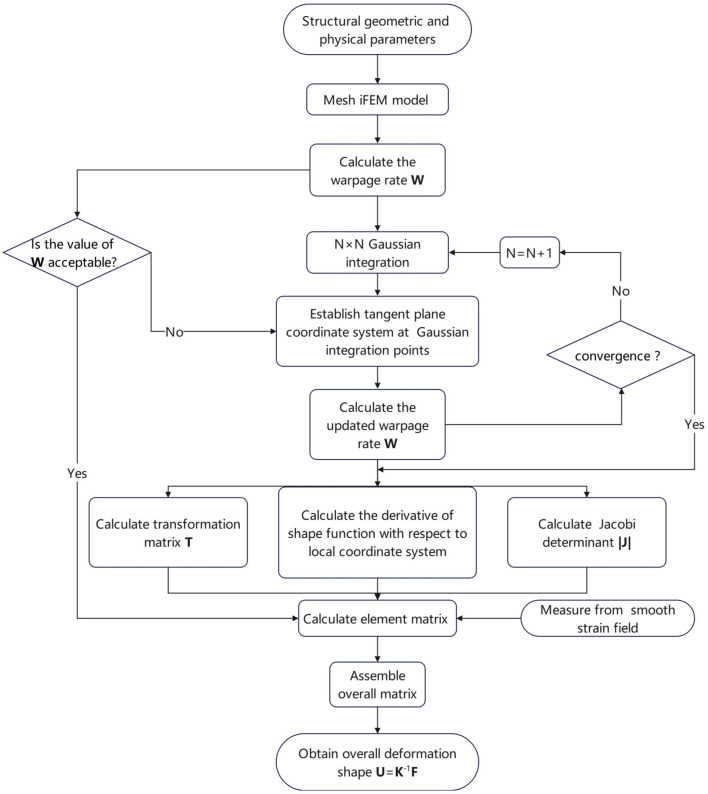
Implementation process of new iFEM algorithm.

In order to write a computer program, we propose a new iFEM algorithm. In conjunction with the new methods we proposed and the conventional iFEM algorithm described before, the new implementation process is shown in Figure 8. 

For ease of presentation, we refer to this new algorithm as iFEM-n for short. In the next section, we verify the validity and accuracy of iFEM-n.

## 3. Numerical Examples 

In this section, the performance of iFEM-n in the displacement reconstruction of curved surfaces will be validated through two numerical examples involving a plate and shell. 

From a numerical calculation perspective, the strain at the Gaussian integration points of each element is relatively straightforward to obtain. The requisite conditions are a sufficiently dense mesh of the geometric model and calculations using FEM to obtain an approximately smooth strain field. This approach allows for the strain at any Gaussian integration point to be determined and used as a measured strain. That is to say, the grids of the iFEM are not synchronised with ABAQUS. Instead, appropriate grids are meshed according to the needs, and the strain required for the iFEM is extracted from the smooth strain field of ABAQUS.

In the event that the materials are assumed to be linear elastic, continuous, and homogeneous, as well as isotropic, all cases are calculated using the software ABAQUS. The strains on the surfaces are extracted as the strains measured by the strain-sensing system after the deformation of the structures. The measured strains can then be inputted into the program written using the new algorithm to achieve the full-field displacement reconstruction of the structures.

In order to ensure that mesh refinement does not excessively interfere with the calculation results, we introduce mesh independence verification for all examples. Furthermore, given the difficulty of meshing some curved structures into regular structured grids [38], we have conducted a verification process to assess the adaptability of iFEM-n to irregular meshes. This involves meshing the model into regular and irregular grids, respectively.

In order to verify the accuracy of iFEM-n, the calculation results will be compared with the calculation results of the iQS4 element, and the calculation results of the finite element software ABAQUS will be used as the reference solution. The results of FEM, iFEM-n, and the iQS4 element are abbreviated as FEM, iFEM-n, and iQS4, respectively.

### 3.1. Twisted Plate

**Figure 9:**
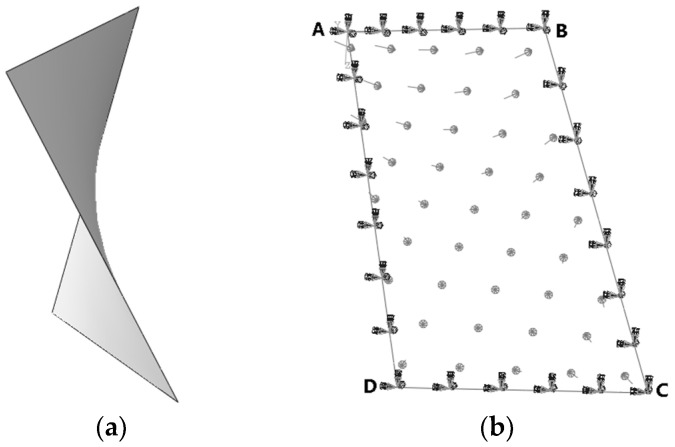
Geometry and loads of the twisted plate. (**a**) Geometry of the twisted plate; (**b**) loads of the twisted plate.

In order to verify the performance of iFEM-n for the displacement reconstruction of irregular plate structures, consider the twisted plate shown in Figure 9a. The modulus of elasticity $E=2\times 10^{11}\;Pa$, Poisson's ratio v=0.3, length L=3.3 m, width b=2.2 m, and thickness t=0.02 m. The twisted plate is rigidly fixed at four sides and is subjected to a pressure $P=1\times 10^{6}\;Pa$, with the direction of pressure directed towards the inner surface of the plate, as shown in Figure 9b. 

**Figure 10:**
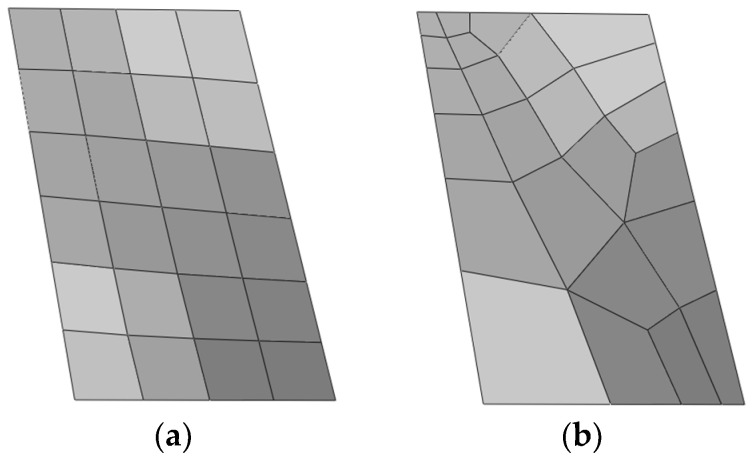
Meshing methods of the twisted plate. (**a**) Regular meshing; (**b**) irregular meshing.

The plate is meshed into regular and irregular grids, respectively. Both edges AB and DC are meshed into 2N elements and both edges BC and AD are meshed into 3N elements. In order to visualise the distinction between the two meshing methods, Figure 10 presents a simple example. Since irregular grids lack a regular number of elements, they are unsuitable for direct representation on the axes. For ease of representation, we abbreviate the number of elements as 2N×3N.

The total displacement $U_{m}$ is defined as:

(14) $$U_{m}=\sqrt{U_{1}^{2}+U_{2}^{2}+U_{3}^{2}}$$

The percent difference $D_{p}$ is defined as:

(15) $$D_{p}^{iFEM-n\;or\;iFEM}=\frac{\left|U_{m}^{FEM}-U_{m}^{iFEM-n\;or\;iFEM}\right|}{U_{m}^{FEM}}\times 100$$

**Figure 11:**
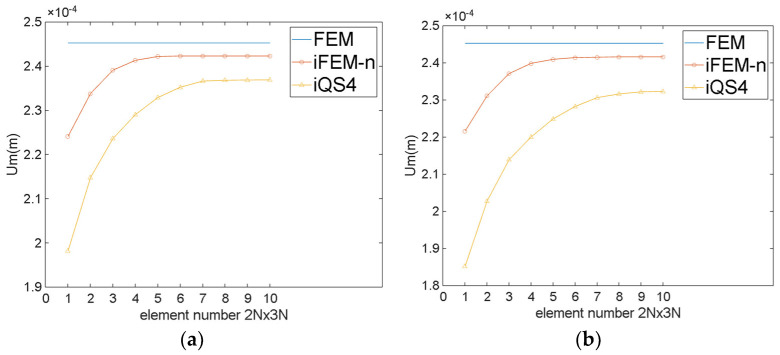
The maximum $U_{m}$ of the twisted plate for different numbers of elements. (**a**) Regular meshing; (**b**) irregular meshing.

Figure 11 and Table 1 demonstrate the maximum $U_{m}$ of the twisted plate for the different number of elements. As can be obviously seen from Figure 11 and Table 1, for regular meshing, iFEM-n converges when the number of elements is 10×15, while iQS4 converges when the number of elements is 14×21. For irregular meshing, iFEM-n converges when the number of elements is 12×18, while iQS4 converges when the number of elements is 18×27. This result indicates that the iFEM-n converges more rapidly than the iQS4. A comparison of Figure 11a,b reveals that the number of elements of iFEM-n increases by approximately 60 when irregular meshing is employed, whereas the number of elements of iQS4 increases by approximately 150. This demonstrates that, even when irregular meshing is utilised, iFEM-n continues to converge at a significantly faster rate than iQS4.

**Figure 12:**
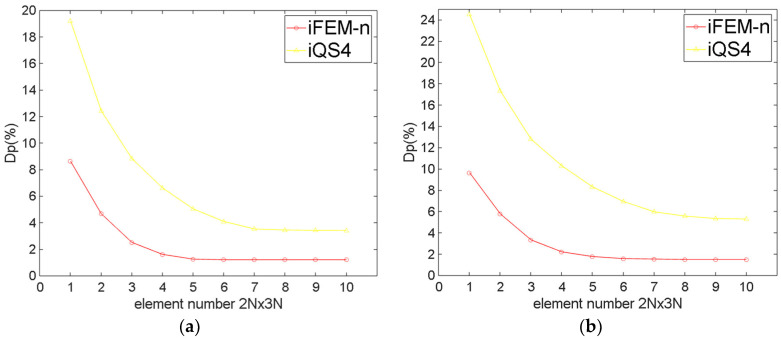
The maximum $D_{p}$ of the twisted plate for different numbers of elements. (**a**) Regular meshing; (**b**) irregular meshing.

Figure 12 and Table 2 show that the maximum $D_{p}$ of the twisted plate for different numbers of elements. It can be seen that for both meshing methods, regardless of the number of elements, the $D_{p}$ of iFEM-n is considerably smaller than for iQS4. Specifically speaking, for regular meshing, when iFEM-n converges, the maximum $D_{p}$ of iFEM-n is 1.20%, while that of iQS4 is 5.04%. For irregular meshing, when iFEM-n converges, the maximum $D_{p}$ of iFEM-n is 1.52%, while that of iQS4 is 6.94%. Comparing the maximum $D_{p}$ of iFEM-n and that of iQS4, it can be seen that not only is the reconstruction accuracy of iFEM-n much higher than that of iQS4, but iFEM-n can also be adapted to irregular grids better than iQS4.

**Figure 13:**
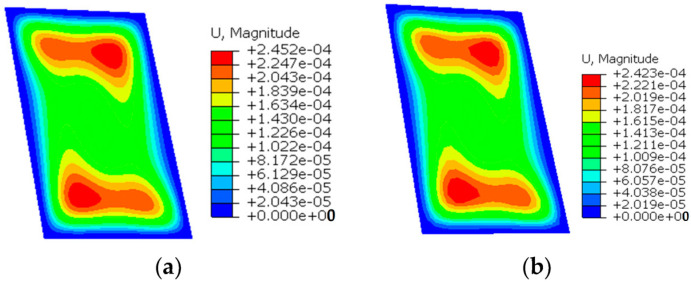

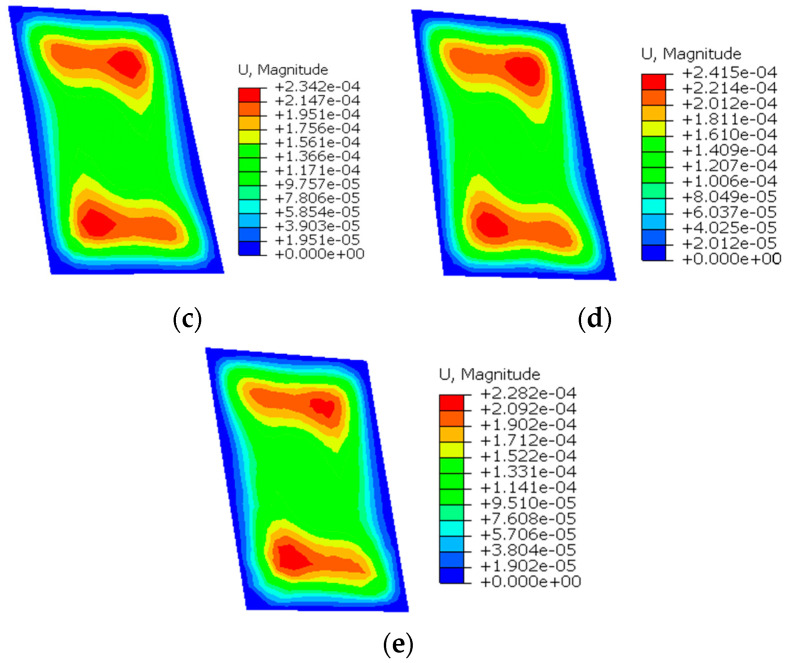
Contour plots of $U_{m}$ for the twisted plate. (**a**) FEM; (**b**) iFEM-n for regular meshing; (**c**) iQS4 for regular meshing; (**d**) iFEM-n for irregular meshing; (**e**) iQS4 for irregular meshing.

Figure 13 illustrates the contour plots of $U_{m}$ of the twisted plate when the number of elements is 10×15 for regular meshing and 12×18 for irregular meshing. It can be observed from the figure that the contour plot of iFEM-n is clearly very similar to the FEM for both meshing methods, while the iQS4 is different compared to the FEM. Moreover, when compared to the regular meshing, the contour plot of iFEM-n remains smooth when irregular meshing is employed, whereas that of iQS4 is uneven. This demonstrates that even when irregular meshing is utilised, iFEM-n is capable of accurately reconstructing the displacement field of the twisted plate.

### 3.2. Hemispherical Shell

**Figure 14:**
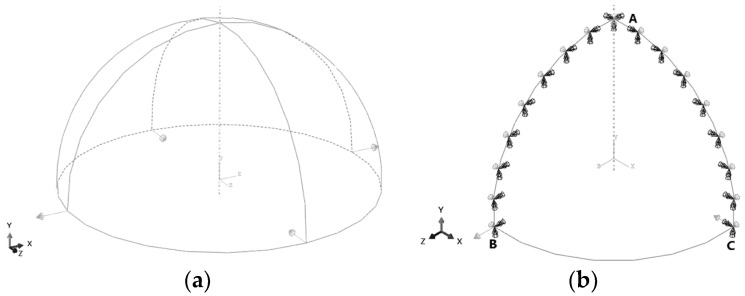
Geometry and loads of the hemispherical shell. (**a**) Full model; (**b**) 1/4 model.

In order to further verify the overall displacement reconstruction capability of iFEM-n for curved shell structures, a hemispherical shell is selected for displacement reconstruction, as shown in Figure 14a. Uniform thickness t=0.02 m, and radius R=1 m. The modulus of elasticity $\;E=2\times 10^{11}$ Pa, Poisson's ratio v=0.3. The hemispherical shell is subjected to two symmetrical pairs of concentrated force loads in the radial direction, with the magnitude of all concentration forces being 1000 N. 

Due to the symmetry of geometry and loading, the 1/4 model (shown in Figure 14b) is utilised for computational analysis and the boundary conditions of the computational model are as follows:

1. Edge AB: $U_{1}=\theta_{2}=\theta_{3}=0$;

2. Edge AC: $U_{3}=\theta_{2}=\theta_{1}=0$;

3. Point A: $U_{2}=0$.

**Figure 15:**
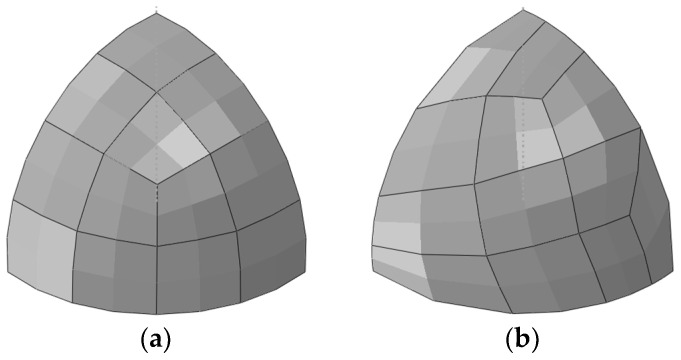
Meshing methods of the hemispherical shell. (**a**) Regular meshing; (**b**) irregular meshing.

The hemispherical shell is meshed into regular and irregular grids, respectively. Both edges AB and AC are meshed into N elements, and edge BC is also meshed into N elements. Figure 15 illustrates an example of the distinction between the two meshing methods. Similarly, the number of elements is abbreviated as N×N.

**Figure 16:**
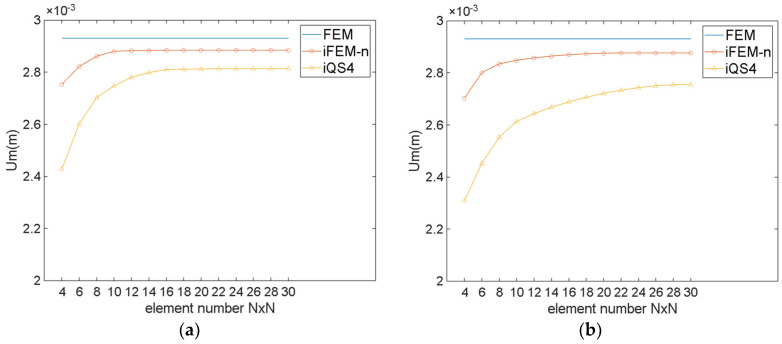
The maximum $U_{m}$ of the hemispherical shell for different numbers of elements. (**a**) Regular meshing; (**b**) irregular meshing.

Figure 16 shows the maximum $U_{m}$ of the hemispherical shell for different numbers of elements. As illustrated in Figure 16, for regular meshing, iFEM-n converges when the number of elements is 12×12, while iQS4 converges when the number of elements is 16×16. For irregular meshing, iFEM-n converges when the number of elements is 16×16, while iQS4 converges when the number of elements is 28×28. This indicates that iFEM-n converges faster than iQS4. A comparison of Figure 16a,b reveals that, in contrast to regular meshing, the number of elements of iFEM-n increases by approximately 80 when irregular meshing is employed, while that of iQS4 increases by approximately 250. This demonstrates that, even when irregular meshing is utilised, iFEM-n continues to demonstrate excellent convergence properties.

**Figure 17:**
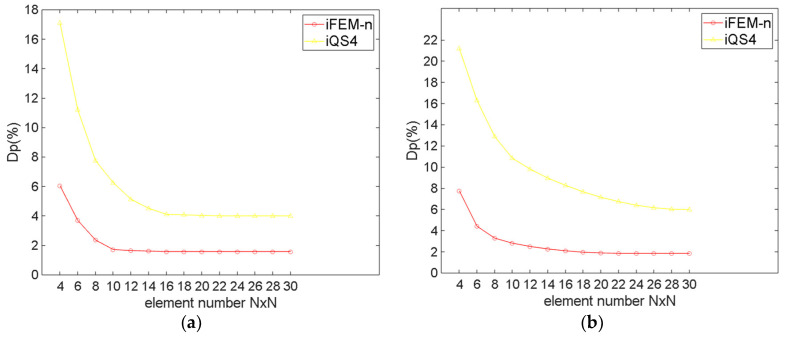
The maximum $D_{p}$ of the hemispherical shell for different numbers of elements. (**a**) Regular meshing; (**b**) irregular meshing.

Figure 17 demonstrates that the maximum $D_{p}$ of the hemispherical shell for different numbers of elements. It can be observed that for both meshing methods, regardless of the number of elements, the $D_{p}$ of iFEM-n is much smaller than for iQS4. Specifically speaking, for regular meshing, when the iFEM-n converges, the maximum $D_{p}$ of iFEM-n is 1.87%, while that of iQS4 is 5.53%. For irregular meshing, when iFEM-n converges, the maximum $D_{p}$ of iFEM-n is 2.23%, while that of iQS4 is 8.32%. Comparing maximum $D_{p}$ of iFEM-n and of iQS4, it can be observed that the reconstruction accuracy of iFEM-n is considerably higher than that of iQS4. Furthermore, iFEM-n is more adept at adapting to irregular grids than iQS4.

**Figure 18:**
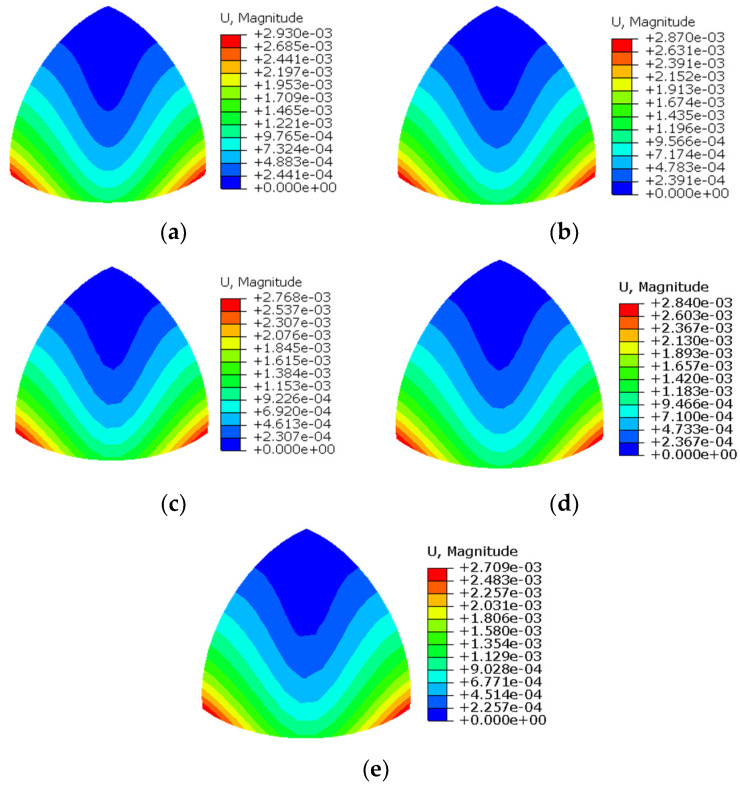
Contour plots of $U_{m}$ for the hemispherical shell. (**a**) FEM; (**b**) iFEM-n for regular meshing; (**c**) iQS4 for regular meshing; (**d**) iFEM-n for irregular meshing; (**e**) iQS4 for irregular meshing.

Figure 18 illustrates the contour plots of $U_{m}$ of the hemispherical shell when the number of elements is 12×12 for regular meshing and 16×16 for irregular meshing. It can be observed from the figure that the contour plot of iFEM-n is clearly very similar to the FEM for both meshing methods, while the iQS4 is different compared to the FEM. Moreover, when compared to the regular meshing, the contour plot of iFEM-n remains smooth when irregular meshing is employed, whereas that of iQS4 is uneven. This demonstrates that even when irregular meshing is utilised, iFEM-n is capable of accurately reconstructing the displacement field of the hemispherical shell.

### 3.3. Summary of This Section 

In this section, the new algorithm proposed in this paper was used to calculate and analyse two typical examples. A comparison of the results of the examples shows that the new algorithm has significantly improved the computational performance of the iQS4 element, regardless of whether the meshing is regular or irregular. The new algorithm is able to effectively improve the computational accuracy, and the results of the computation converges faster than iQS4.

Therefore, it can be concluded that the new algorithm proposed in this paper outperforms the conventional iQS4 element in the deformation reconstruction of irregular or large curvature surface shell structures, and can realise the deformation reconstruction with high accuracy. This new algorithm is designated as iFEM-H.

## 4. Practical Engineering Applications 

The harsh marine environment and strong climatic conditions are likely to cause damage to the ship structure. The ship must not only bear long-term cyclic loads from continuous waves, but also bear short-term extreme loads such as huge waves, rainstorms, strong winds, and sea earthquakes. Furthermore, contact between seawater and ship materials (mostly high-strength steel) can lead to rapid corrosion and erosion, resulting in a decrease in thickness [25]. Consequently, it is imperative to implement precise SHM on ships. Given the fact that containerships represent a highly representative marine structure, this section will utilise the proposed iFEM-H for the shape sensing of a containership.

**Figure 19:**
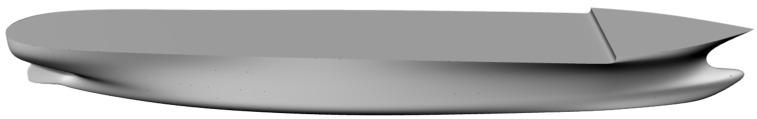
Geometric model of the containership.

**Figure 20:**
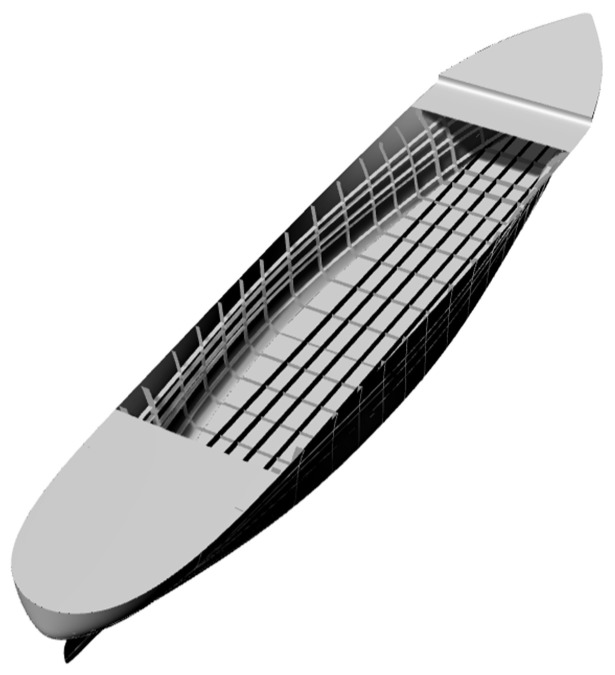
Internal structure of the containership.

Figure 19 shows a representative containership. In order to simulate actual working conditions as closely as possible, we employed the ABAQUS software to calculate the containership model with an internal structure (shown in Figure 20). Subsequently, we extracted the strain values calculated by FEM as the measured strain. As the focus of this section is to demonstrate the practicality of the iFEM-H for curved structures, in reality, it is sufficient to perform precise shape sensing on the shell of the containership. Consequently, when utilising the iFEM-H, it is sufficient to consider the geometric model of the shell and to mesh it. By measuring the strain of the shell, it is possible to achieve a full field deformation reconstruction of the shell.

For clarity, only one global coordinate system (X,Y,Z) serves as containership frame of reference, with its origin (0,0,0) located at the still waterline and aligned vertically with the ship's centre of gravity. The X, Y, and Z axes of the coordinate system point out the bow, portside, and opposite direction of gravity, respectively. According to the global coordinate system, when the containership is loaded at its design draught, the containership has the general particulars listed in Table 3. 

**Figure 21:**
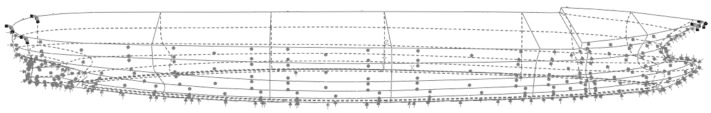
Loads of the containership.

**Figure 22:**
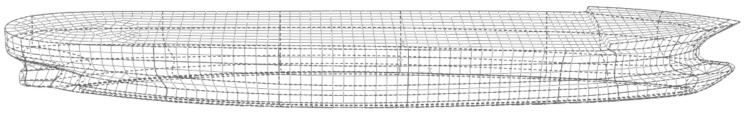
Grids of the containership.

For simplicity, all of the structural components have been designed to have a uniform thickness of t=30 mm and they are made of steel with an elastic modulus of E=210 GPa and Poisson's ratio of ν=0.3. Assuming that the containership is docked at the harbour, subject to hydrostatic pressure only, the reinforced parts of the bow and stern can be regarded as non-deformable regions and the boundary conditions are set to be rigidly fixed, as shown in Figure 21. The containership is meshed into 5669 inverse elements, as shown in Figure 22.

**Figure 23:**
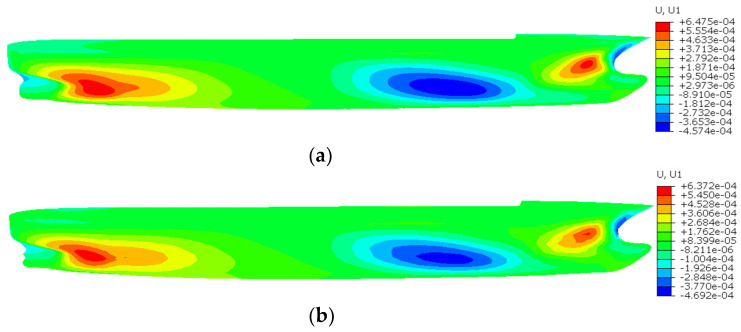
Contour plots of $U_{1}$ for the containership. (**a**) FEM; (**b**) iFEM-H.

**Figure 24:**
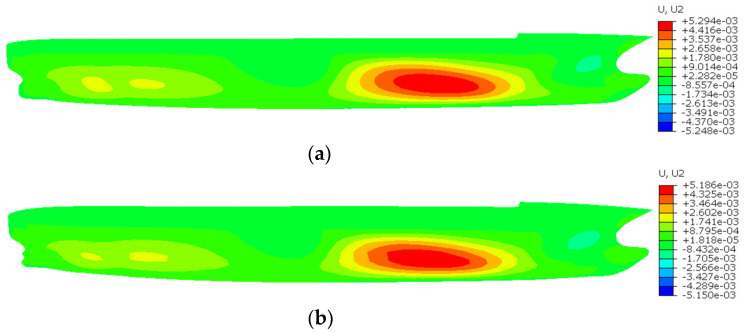
Contour plots of $U_{2}$ for the containership. (**a**) FEM; (**b**) iFEM-H.

**Figure 25:**
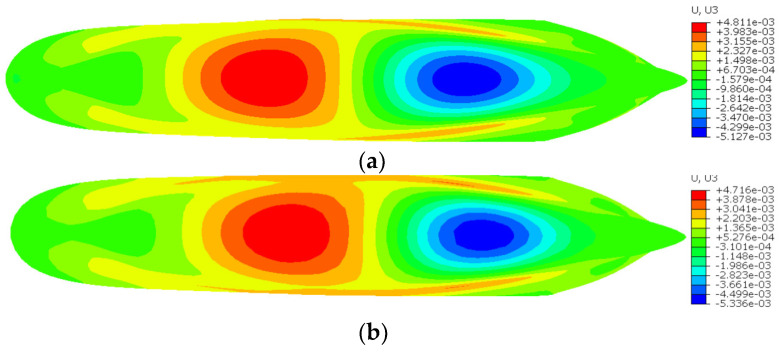
Contour plots of $U_{3}$ for the containership. (**a**) FEM; (**b**) iFEM-H.

Figure 23, Figure 24 and Figure 25 show the contour plots of $U_{1}$, $U_{2}$, and $U_{3}$ for the containership, respectively. Table 4 shows the maximum $U_{1}$, $U_{2}$, and $U_{3}\;$and the percentage difference between FEM and iFEM-H for the containership. It is evident that all contour plots of the iFEM-H exhibit a high degree of similarity to those of the FEM. Specifically, iFEM-H produces a maximum $U_{1}$ with a percent difference of 1.59% relative to the FEM maximum $U_{1}$; iFEM-H produces a maximum $U_{2}$ with a percent difference of 2.38% relative to the FEM maximum $U_{2}$; and iFEM-H produces a maximum $U_{3}$ with a percent difference of 1.97% relative to the FEM maximum $U_{3}$.

As illustrated in Figure 23, Figure 24 and Figure 25 and Table 4, the utilisation of iFEM-H for the purpose of the structural health monitoring of the containership results in a small displacement error, with the overall deformation trend exhibiting a high degree of consistency. This outcome is deemed to be within the acceptable limits of engineering practice. Given the highly representative nature of containerships as marine structures, it can be considered that the iFEM-H is capable of accurately sensing the shape of some marine structures with complex surfaces.

## 5. Conclusions

This article presents a high-precision inverse finite element method (iFEM-H) to address the crucial need for monitoring the health of ship structures. The key contributions of this research can be summarised as follows: 

(a) A novel algorithm for iFEM has been devised, based on a simple alteration to the underlying computational principles. Firstly, a method for continuously correcting the transformation matrix has been proposed. This method establishes a local coordinate system on the tangent plane and, further, on the Gaussian integration points. Subsequently, a method for directly calculating element stiffness integration using three-dimensional spatial coordinates has been developed. The combination of the aforementioned novel computational methods with the fundamental iFEM formulation has led to the development of the new iFEM algorithm.

(b) Numerical examples demonstrate that the novel iFEM algorithm outperforms the iQS4 element in terms of both computational accuracy and the speed of convergence in the reconstruction of curved shell structures. Furthermore, the new iFEM algorithm is more suitable for irregular grids, which is crucial when solving complex surface problems. Therefore, we propose to designate this new algorithm as iFEM-H. 

(c) The iFEM-H was employed for the displacement monitoring of a containership. Measured strain data were obtained from ABAQUS to represent the deformation of the containership in a realistic marine environment. The numerical results demonstrate that the iFEM-H has the capacity to achieve the precise shape sensing of some marine structures with complex surfaces. 

## Figures and Tables

**Table 1:** The maximum $U_{m}$ of the twisted plate for different numbers of elements.

Element Number 2N × 3N	Maximum U_m_(m)				
	FEM	Regular Meshing	Irregular Meshing		
		iFEM-n	iQS4	iFEM-n	iQS4
1	2.452 × 10^−4^	2.241 × 10^−4^	1.981 × 10^−4^	2.216 × 10^−4^	1.851 × 10^−4^
2		2.338 × 10^−4^	2.147 × 10^−4^	2.311 × 10^−4^	2.027 × 10^−4^
3		2.391 × 10^−4^	2.236 × 10^−4^	2.370 × 10^−4^	2.139 × 10^−4^
4		2.413 × 10^−4^	2.290 × 10^−4^	2.398 × 10^−4^	2.200 × 10^−4^
5		2.420 × 10^−4^	2.342 × 10^−4^	2.409 × 10^−4^	2.248 × 10^−4^
6		2.421 × 10^−4^	2.351 × 10^−4^	2.414 × 10^−4^	2.282 × 10^−4^
7		2.422 × 10^−4^	2.355 × 10^−4^	2.414 × 10^−4^	2.305 × 10^−4^
8		2.422 × 10^−4^	2.357 × 10^−4^	2.415 × 10^−4^	2.316 × 10^−4^
9		2.422 × 10^−4^	2.358 × 10^−4^	2.415 × 10^−4^	2.321 × 10^−4^
10		2.422 × 10^−4^	2.358 × 10^−4^	2.415 × 10^−4^	2.322 × 10^−4^

**Table 2:** The maximum $D_{p}$ of the twisted plate for different numbers of elements.

Element Number 2N × 3N	Maximum D_p_ (%)			
	Regular Meshing	Irregular Meshing		
	iFEM-n	iQS4	iFEM-n	iQS4
1	8.62	19.19	9.64	24.50
2	4.67	12.43	5.77	17.32
3	2.50	8.82	3.32	12.78
4	1.60	6.61	2.20	10.28
5	1.21	5.04	1.76	8.30
6	1.20	4.09	1.54	6.94
7	1.20	3.52	1.53	5.96
8	1.20	3.44	1.52	5.56
9	1.20	3.42	1.52	5.33
10	1.20	3.42	1.52	5.29

**Table 3:** General particulars of the containership.

General Particular	Value	Unit
Length between perpendiculars	217.2	m
Breadth	39.6	m
Depth	24.9	m
Design draught	14.9	m

**Table 4:** Maximum $U_{1}$, $U_{2}$, and $U_{3}$ and percentage difference between FEM and iFEM-H for the containership.

Displacement Component	Displacement (m)		Difference (%)
	FEM	iFEM-H	
U1	6.475 × 10^−4^	6.372 × 10^−4^	1.59
U2	5.294 × 10^−3^	5.186 × 10^−3^	2.38
U3	4.811 × 10^−3^	4.716 × 10^−3^	1.97

## Data Availability

Data are contained within the article.
